# 46,XY,r(8)/45,XY,−8 Mosaicism as a Possible Mechanism of the Imprinted Birk-Barel Syndrome: A Case Study

**DOI:** 10.3390/genes11121473

**Published:** 2020-12-09

**Authors:** Anna A. Kashevarova, Tatyana V. Nikitina, Larisa I. Mikhailik, Elena O. Belyaeva, Stanislav A. Vasilyev, Mariya E. Lopatkina, Dmitry A. Fedotov, Elizaveta A. Fonova, Aleksei A. Zarubin, Aleksei A. Sivtsev, Nikolay A. Skryabin, Lyudmila P. Nazarenko, Igor N. Lebedev

**Affiliations:** 1 Research Institute of Medical Genetics, Tomsk National Research Medical Center (NRMC), Ushaika Street 10, 634050 Tomsk, Russia; t-nikitina@medgenetics.ru (T.V.N.); elena.belyaeva@medgenetics.ru (E.O.B.); Stanislav.vasilyev@medgenetics.ru (S.A.V.); maria.lopatkina@medgenetics.ru (M.E.L.); feddmi.mg@gmail.com (D.A.F.); fonova.elizaveta@medgenetics.ru (E.A.F.); aleksei.zarubin@medgenetics.ru (A.A.Z.); sivtsev.alexey@medgenetics.ru (A.A.S.); nikolay.skryabin@medgenetics.ru (N.A.S.); ludmila.nazarenko@medgenetics.ru (L.P.N.); igor.lebedev@medgenetics.ru (I.N.L.); 2 Regional Clinical Center for Specialized Medical Care, 690091 Vladivostok, Russia; larisa053@mail.ru

**Keywords:** ring chromosome 8, invdupdel(8p), Birk-Barel syndrome, *KCNK9*, imprinting

## Abstract

Ring chromosome 8 (r(8)) is one of the least frequent ring chromosomes. Usually, maternal chromosome 8 forms a ring, which can be lost from cells due to mitotic instability. The 8q24 region contains the imprinted *KCNK9* gene, which is expressed from the maternal allele. Heterozygous *KCNK9* mutations are associated with the imprinting disorder Birk-Barel syndrome. Here, we report a 2.5-year-old boy with developmental delay, microcephaly, dysmorphic features, diffuse muscle hypotonia, feeding problems, motor alalia and noncoarse neurogenic type of disturbance of muscle electrogenesis, partially overlapping with Birk-Barel syndrome phenotype. Cytogenetic analysis of lymphocytes revealed his karyotype to be 46,XY,r(8)(p23q24.3)[27]/45,XY,−8[3]. A de novo 7.9 Mb terminal 8p23.3p23.1 deletion, a 27.1 Mb 8p23.1p11.22 duplication, and a 4.4 Mb intact segment with a normal copy number located between them, as well as a 154-kb maternal *LINGO2* gene deletion (9p21.2) with unknown clinical significance were identified by aCGH + SNP array. These aberrations were confirmed by real-time PCR. According to FISH analysis, the 8p23.1-p11.22 duplication was inverted. The ring chromosome originated from maternal chromosome 8. Targeted massive parallel sequencing did not reveal the *KCNK9* mutations associated with Birk-Barel syndrome. Our data allow to assume that autosomal monosomy with inactive allele of imprinted gene arising from the loss of a ring chromosome in some somatic cells may be an etiological mechanism of mosaic imprinting disorders, presumably with less severe phenotype.

## 1. Introduction

Ring chromosome 8 (r(8)) is a rare abnormality. It accounts for 4% of all rings [1]. Patients with nonsupernumerary r(8) are characterized by short stature, microcephaly, and variable intellectual disability, without major anomalies [2]. A mosaic karyotype with a monosomy 8 cell line due to r(8) loss is frequently observed in such individuals.

One of the main mechanisms of ring chromosome formation is through invdupdel rearrangements, which are then stabilized by circularization [3]. Duplications just proximal to the deletion breakpoint account for approximately 21% of ring chromosome cases [4]. The first invdupdel(8p) case was presented by Weleber and colleagues in 1976 [5]. The incidence of invdupdel(8p) in the general population is estimated as 1 per 10,000–30,000 liveborn infants [6]. The rearrangement consists of a terminal deletion followed by an inverted duplication. There may be an intact segment between the duplicated and deleted regions if one of the parents carries an inversion. The proximal 8p23 region is predisposed to polymorphic inversion between two olfactory receptor gene clusters, which increases susceptibility to 8p rearrangements during meiotic chromosome segregation [7,8,9]. The frequency of this inversion is 27% in the normal Japanese population and 26% in the European population [7,10]. To date, only 11 patients with invdupdel(8p) have been described [6,8,11]. The mothers of all these patients were heterozygous carriers of paracentric 8p23.1 inversion. Therefore, it has been assumed that this aberration appears during maternal meiosis.

The 8q24.3 region contains the imprinted *KCNK9* gene (OMIM [12] 605874). This gene belongs to the family of two-pore domain potassium channel genes, which regulate the resting membrane potential and influence action potential duration and neuron firing frequency [13]. This gene encodes the TASK-3 protein, which in humans is expressed in the brain with particularly high levels in the cerebellum [14], where it participates in the development and maturation of cerebellar neurons [15]. Ruf and colleagues showed the *KCNK9* expression from the maternal allele in fetal human brain and adult mouse brain [16].

Heterozygous mutations in the *KCNK9* gene on the maternal allele led to Birk-Barel syndrome (BBS; OMIM 612292) [17,18,19]. This syndrome is characterized by congenital central hypotonia, severe feeding difficulties, delayed development/intellectual disability, and characteristic dysmorphic features. To date, approximately two dozen individuals with a molecularly confirmed diagnosis have been reported [17,18,19].

There are five major causes of imprinting disorders: deletions or duplications of the imprinted gene, uniparental disomy (UPD), point mutations in the imprinted gene, and mutations in imprinting control centers. One can assume that autosomal monosomy with inactive allele of imprinted gene arising from the loss of a ring chromosome in some somatic cells may be an additional etiological mechanism of mosaic imprinting disorders, presumably with less severe phenotype. To date, there is only one description of a patient with Prader-Willi syndrome (PWS) and a non-mosaic r(15) of paternal origin in blood culture [20]. The authors suggest that this may be due to partial inactivation of the PWS region due to position effects or that the ring might have been lost in some tissues, leading to an absence of the imprinted genes on paternal chromosome 15. It has been observed that several patients with r(15) had multiple PWS features: short stature, low birth weight, hypotonia, small hands and feet, intellectual disability, and others. It seems that the combination of features and their severity may depend on the proportion of cells with autosomal monosomy due to ring chromosome loss and their tissue distribution.

Here, we report a patient with mosaic karyotype presented by two cell clones with r(8) of maternal origin and monosomy 8 due to the loss of the ring chromosome. The patient had hypotonia and a noncoarse neurogenic type of disturbance of muscle electrogenesis (ENMG neurogenic pattern), which with normal conductivity along the motor fibers of peripheral nerves, does not exclude one of the genetic variants of spinal muscular atrophy (SMA) or atypical SMA and may be consistent with clinical features of BBS.

### Case Presentation

The patient, a boy 1 year and 4 months old, was born from a second pregnancy. The first pregnancy ended in miscarriage. The patient was a single birth to nonconsanguineous, healthy parents. His pedigree was unremarkable. Written informed consent was obtained from the parents of the patient for the publication of the clinical data and pictures. The study was approved by the local Research Ethics Committee of the Research Institute of Medical Genetics, Tomsk NRMC (no. 106 from 27 June 2017).

The patient was born at the 38th week of gestation. His birth weight was 2720 g (3–10th centile), birth length was 48 cm (10th centile), head circumference was 31 cm (<3rd centile), and chest circumference was 31 cm (3rd centile). His Apgar score was 8. Preauricular tag of the left ear, curvature of the nasal septum to the left, and pectus excavatum of the right foot were observed at birth. The early development of the child showed pronounced delays.

When the boy was 1 year old, echoencephalography showed ventriculomegaly, and echocardiography revealed a small heart abnormality, patent foramen ovale. When the patient was 1 year and 4 months of age, his height was 80 cm (75th centile), his weight was 8.6 kg (3rd centile), and his head circumference was 40.5 cm (<3rd centile). His constitution was disproportionate and asthenic. There was a severe delay in neuropsychological development. The boy could not stand up or remain standing on his own and walked only with bilateral support; he did not sit on his own but remained seated when already sitting. His gait was hesitant, and he slightly tightened his left leg (extra step). The patient held his posture weakly, i.e., his back was rounded. He did not crawl or turn over while lying on his stomach, and his support when on his hands was weak. A more detailed clinical description of the patient at the time of cytogenetic analysis is shown in Table 1.

**Figure 1:**
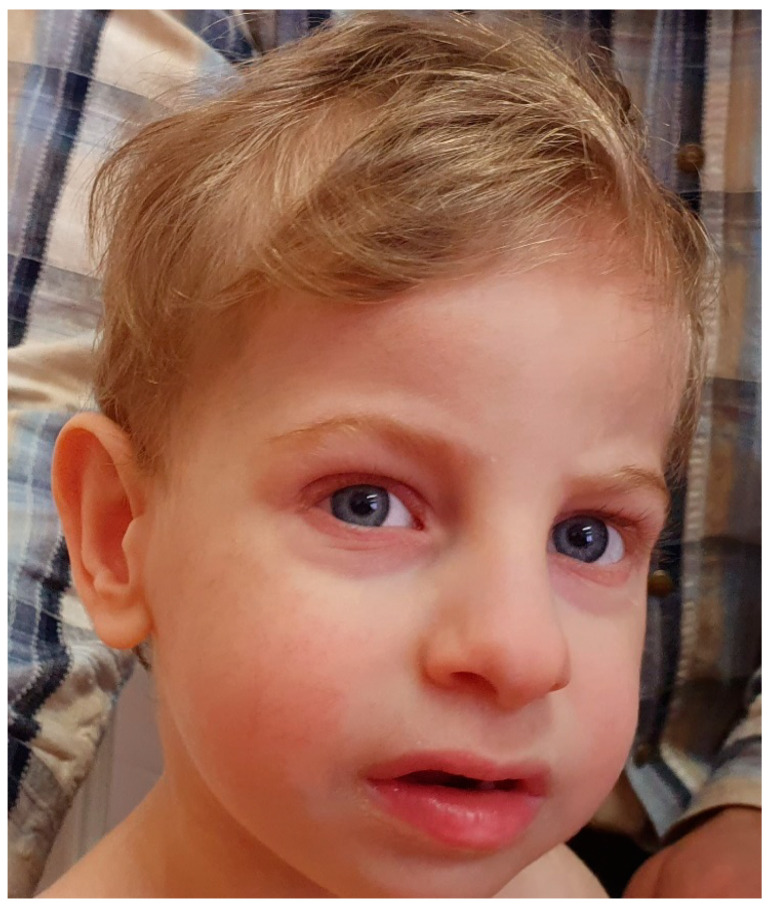
The patient at 2.5 years of age (note dolichocephaly, narrowing of the skull in the temporal areas, elongated face, bushy eyebrows, long eyelashes, macrotia, high and narrow nasal bridge with a broad nasal tip, short and broad philtrum, open mouth, and thick lips).

At the time of last examination, the boy was 2.5 years old (Figure 1). He had dolichocephaly, narrowing of the skull in the temporal areas, an elongated face, bushy eyebrows, long eyelashes, conjunctivitis, abnormal ears with a preauricular tag of the left ear, macrotia, a high and narrow nasal bridge with a broad nasal tip, a short and broad philtrum, hypotonia of the mandible, micrognathia, an open mouth, thick lips, widely spaced teeth, and motor alalia. The child swallowed only liquid food. Gastroesophageal reflux was constantly present. The tone of the chewing and temporal muscles was reduced, as were facial movements. There were problems with fine motor skills. The patient walked with support only, and the gait was abnormal. Contact with the child was difficult, as there were periods of increased excitability, and the child was unemotional.

## 2. Materials and Methods

### 2.1. Materials

Peripheral blood was collected in tubes containing EDTA for molecular genetic analyses and heparin for banding cytogenetics.

### 2.2. Cytogenetic Analyses

Banding cytogenetic analysis was performed based on GTG-banded metaphases from peripheral blood lymphocytes from the patient at a 400-band resolution.

A neurologist diagnosed diffuse muscle hypotonia and moderate proximal muscle weakness. According to electroneuromyography (ENMG), the boy had a noncoarse neurogenic type of disturbance of muscle electrogenesis (ENMG neurogenic pattern), which, with normal conductivity along the motor fibers of peripheral nerves, does not exclude one of the genetic variants of spinal muscular atrophy (SMA) or atypical SMA.

### 2.3. Array-Based Comparative Genomic Hybridization (aCGH) Analyses

aCGH was performed using the SurePrint G3 Human CGH + SNP Microarray Kit (4 × 180 K) (Agilent Technologies, Santa Clara, CA, USA), according to the manufacturer’s recommendations. Labelling and hybridization of the patient and reference DNA (#5190-3796, Human Reference DNA, Agilent Technologies) were performed using enzymatic labelling and hybridization protocols (v. 7.5, Agilent Technologies). Array images were acquired with an Agilent SureScan Microarray Scanner (Agilent Technologies). Data analysis was performed using CytoGenomics software (v. 3.0.6.6) (Agilent Technologies) and the publicly available Database of Genomic Variants (DGV) [27].

### 2.4. Confirmation of Copy Number Variations Using Quantitative Real-Time PCR

Target sequences within the deleted chromosomal regions 8p23.3-p23.1 and 9p21.1 and specific amplification primers for quantitative real-time PCR assays were selected using Primer 3 software [28] (Table S1). The presence of 8p23.3-p23.1 and 9p21.1 microdeletions was tested using genomic DNA from peripheral blood lymphocytes from the patient and his parents using the AriaMx Real-Time PCR System (Agilent Technologies). Control genomic DNA was obtained from the peripheral blood lymphocytes of a healthy donor. Written informed consent was obtained from the donor. The control gene was *HEXB*, which encodes the β subunit of hexosaminidase and is located at 5q13 (Table S1). Real-time PCR was conducted using 25 ng of DNA (10 ng/μL), 2.5 μL (1 μM/L) of forward and reverse primers, 12.5 μL of 2× BioMaster HS-qPCR SYBR Blue (BioLabMix, Novosibirsk, Russia), and RNase-free water to 20 μL (per one well). The real-time PCR conditions were as follows: initial incubation at 95 °C for 10 min followed by 40 cycles of 15 s at 95 °C, 30 s at 60 °C, and 30 s at 72 °C. Three technical replicates were run for each sample. The obtained values of C_T_ for test and reference (control) DNA amplification with primers for test and reference genes were analyzed using the following: average value for C_T_, logQT test primer = (Ct test DNA − CT reference DNA)/slope, (logQT test primer − log QT control primer), and fold change = 10^logQT test primer − logQT control primer^. Fold change values were used to build chart.

### 2.5. Fluorescence In Situ Hybridization (FISH)

FISH was performed using PCR-based probes for the distal (*TUSC3* gene) and proximal (*UNC5D* gene) regions of the 8p23.1–p11.22 duplication in lymphocytes from the proband following the standard protocol. *E. coli* clones carrying plasmids with inserted centromere-specific α-satellite DNA sequences pZ8.4 were kindly provided by Professor M. Rocchi (Resources for Molecular Cytogenetics, Institute of Genetics, Bari, Italy). The probes for the *TUSC3* and *UNC5D* genes were generated using a long-range PCR kit (BioLabMix, Novosibirsk, Russia) (Table S1) [29]. Probes TUSC3 and UNC5D were labelled with TAMRA-dUTP and Fluorescein-dUTP (BioSan, Novosibirsk, Russia), respectively.

### 2.6. Targeted Massive Parallel Sequencing

DNA was extracted from blood samples of the patient and his parents. The regions flanking the exons of the *KCNK9* gene were amplified by PCR using self-designed primers (Table S2). PCR was conducted using 1 μL DNA (initial concentration 100–200 ng/μL), 1 μL of 2.5 μmol/L primers, 12.5 μL BioMaster LR HS-PCR-Color (2×), and sterilized distilled water up to 25 μL per one sample.

Based on amplified targeted regions, libraries were created using a NextEra XT DNA Sample Preparation Kit (24 Samples) (Illumina Inc., San Diego, CA, USA) and NextEra XT Index Kit v2 Set A (Illumina Inc.). Fragment concentration was determined using a Qubit 3 Fluorometer (Thermo Fisher, Lenexa, KS, USA) with dsDNA HS Assay Kit (Thermo Fisher) and mean fragment size was determined using a High Sensitivity D1000 ScreenTape (Agilent Technologies, Santa Clara, CA, USA). Libraries were sequenced on a MiSeq desktop sequencer using MiSeq Reagent Micro Kit, v2 (300 cycles) (Illumina Inc.).

Bioinformatic analysis was carried out using the GATK4 software package according to best practices for discovery Germline short variant (SNPs + Indels) [30].

Read alignment was performed on 38 reference assembly of the human genome (hg38).

Quality control of readings was carried out using FastQC, Qualimap, Bcftools, and MultiQC software packages. Annotation of the detected variants was carried out by the Annovar using databases RefSeq, dbSNP150, gnomAD, Clinvar (version from 2020.03.16), SIFT, and PolyPhen2 [31].

The molecular cytogenetic and molecular genetic studies were performed at the Core Medical Genomics Facility of the Tomsk National Research Medical Center (NRMC) of the Russian Academy of Sciences using the resources of the biocollection “Biobank of the population of Northern Eurasia” of the Research Institute of Medical Genetics, Tomsk NRMC.

## 3. Results

**Figure 2:**
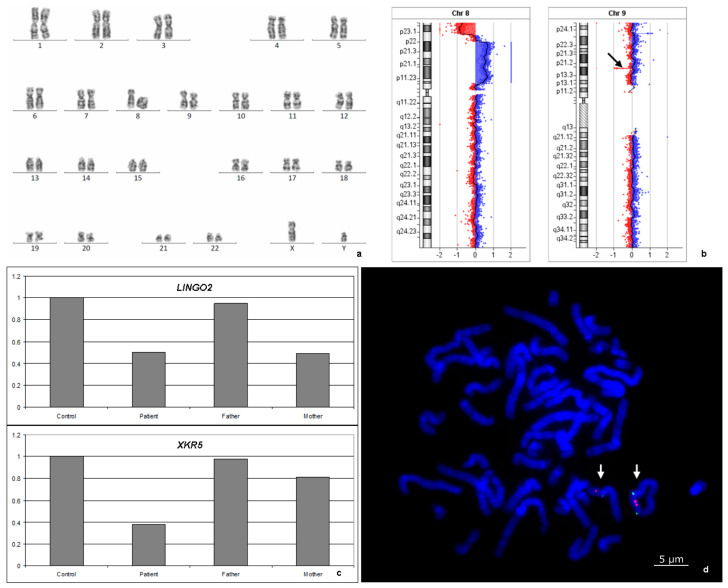
Genetic studies: (**a**) banding cytogenetic analysis: ring chromosome 8; (**b**) an aCGH image of chromosomes 8 and 9 in the lymphocytes of the patient (black arrow indicates del9p21.1); (**c**) confirmation of deletions by quantitative real-time PCR analysis (*LINGO2*—del9p21.1, *XKR5*—del8p23.3–p23.1; X axis are the individuals, Y axis is the multiplicity of differences in the copy number of the DNA within the investigated region compared to the control); and (**d**) FISH analysis using PCR-based probes for *UNC5D* (green) and *TUSC3* (red) in the cultured lymphocytes of the proband. Left white arrow indicates normal chromosome 8, and right white arrow indicates ring chromosome 8.

G-band karyotyping of peripheral lymphocytes showed a 46,XY,r(8)(p23q24.3)[27]/45,XY,−8[3] karyotype for the patient (Figure 2a). FISH analysis detected 24% monosomic cells and 14.6% micronuclei, including 10% centromere-positive micronuclei. aCGH using an Agilent 180K + SNP microarray revealed a 7.9 Mb 8p23.3p23.1 deletion, a 27.1 Mb 8p23.1p11.22 duplication, a 4.4 Mb intact normal copy number segment between them, and a monogenic 154-kb 9p21.1 deletion: arr[hg19]8p23.3p23.1(191530_8079920)×1,8p23.1p11.22(12467484_39587538)×3,9p21.1(28604283_28758185)×1 (Figure 2b). The 9p21.1 microdeletion involved only the *LINGO2* gene. The microdeletion was confirmed via quantitative real-time PCR analysis and was shown to be inherited from the healthy mother (Figure 2c). LINGO2 belongs to the family of leucine-rich repeat and IgG-like domain proteins. The clinical significance of this deletion remains unclear.

According to SNP array analysis, r(8) originated from maternal chromosome 8. The inverted orientation of the duplication was confirmed by FISH (Figure 2d). The combination of inverted 8p23.1p11.22 duplication and terminal 8p23.3p23.1 deletion is denoted as invdupdel(8p).

Finally, targeted massive parallel sequencing of DNA sample obtained from peripheral blood lymphocytes of our patient did not identify either of the mutations in the *KCNK9* gene associated with BBS (c.770G>A, c.770G>C, or c.710C>A) [17,19].

## 4. Discussion

We present a patient with a severe phenotype, r(8) and monosomy 8, determined by banding cytogenetics. aCGH and FISH revealed the following combination of chromosomal abnormalities: del8p23.3p23.1 (7.9 Mb), invdup8p23.1p11.22 (27.1 Mb), del9p21.1 (154 kb), and monosomy for chromosome 8 in 24% of cells.

Several dozen individuals with 8p terminal deletions of variable size have been described so far. Their common features included developmental delay/intellectual disability (DD/ID), growth retardation, microcephaly, mild dysmorphic features (bilateral epicanthal folds, upslanting palpebral fissures, and ear abnormalities), widely spaced nipples, hypospadias, congenital heart defects, congenital diaphragmatic hernia, seizures, and neurobehavioral disorders [32,33,34,35]. The more distal deletion 8p23.2 to 8pter has been reported as a critical region associated with DD/ID, seizures, and neurobehavioral problems (autism spectrum disorders (ASD) and attention-deficit hyperactivity disorder (ADHD)) [34,36,37]. Of the abovementioned phenotypes, the index patient has DD, microcephaly, and neurobehavioral disorders. When the boy was 1 year old, ventriculomegaly and patent foramen ovale were identified.

**Figure 3:**
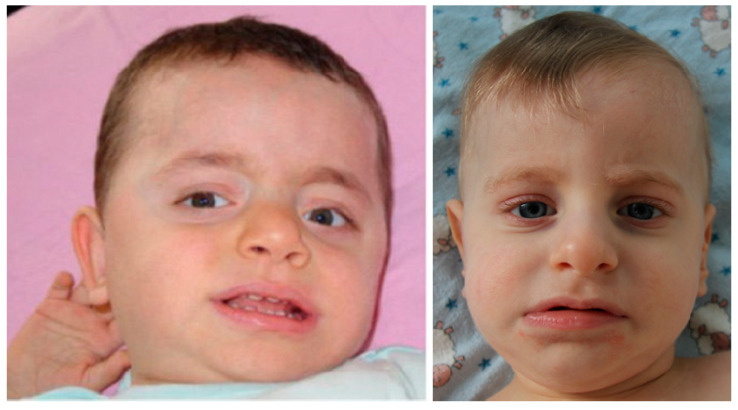
The patient with 46,XY,dup(8)(pter->p23.3::p12->p23.3::p23.3->qter) karyotype (**left**), 1 year and 5 months old [44], and the index patient from the given study with arr[hg19] 8p23.3p23.1(191530_8079920)×1,8p23.1p11.22(12467484_39587538)×3 karyotype (**right**), 1 year and 4 months old. Note similar facial features.

Several types of 8p interstitial partial duplications are known: inverted, direct distal, involving the 8p23.1 region, and direct proximal duplications. Inverted duplications are quite common rearrangements, leading to a recognizable syndrome: some dysmorphic features, severe DD, structural brain anomalies (e.g., corpus callosum agenesis) and hypotonia in childhood (Figure 3) [38]. In adults, spastic paraplegia and orthopedic problems are observed [39]. The 8p21p22 region is suggested to be critical for the described phenotype [39,40]. In all cases of invdup(8p) where a concomitant small terminal deletion has been looked for, it was detected [39,41,42,43]. Comparing the clinical phenotypes of isolated terminal deletions and inverted duplications, possibly in combination with terminal deletions, the common feature is DD, while growth retardation, microcephaly, heart defects, diaphragmatic hernia, and seizures are typical for isolated deletions and structural brain anomalies, hypotonia, spastic paraplegia, and orthopedic problems for inverted duplications. Undoubtedly, the phenotype is affected by the size and gene content of the deletions and duplications. The patient described in this study with invdupdel(8p) had some clinical features of both deletions and duplications, i.e., DD, microcephaly, minor heart defects, and hypotonia. Meanwhile, he also had signs not typical for either: tall stature and feeding problems. In our patient, the invdupdel(8p) rearrangement induced circularization of the abnormal chromosome 8, which was further lost in a portion of cells due to r(8) mitotic instability.

To date, only eight patients with r(8) have been described in the literature (Table 1). Among them is one familial case [2]. The authors give clinical and cytogenetic descriptions of the proband and his mother, both with r(8) mosaic karyotypes, and provide some phenotypic features of the proband’s uncle and grandmother, both with short stature and microcephaly, for whom cytogenetic examination was not possible. For five patients out of the eight with mosaic karyotypes, monosomy 8 cell line, normal karyotype, and double rings were observed. Where the origin of the ring was determined, it was always maternal, i.e., when the ring was lost, the single paternal homologue remained or even doubled, leading to karyotype correction. The karyotype correction occurs more actively in fibroblasts, and the normal clone becomes more represented over time. Gradek and colleagues showed an increase in the 46,XY clone from 0% to 23% in patient lymphocytes from 9 to 19 years of age. They also determined the paternal isodisomy for chromosome 8 in 46,XY cells [26]. In our patient, a 46,XY,r(8)(p23q24.3)[27]/45,XY,−8[3] karyotype was shown by G-banding cytogenetics, and FISH analysis detected 24% of 45,XY,−8 cells among lymphocytes. We also observed a high proportion of micronuclei in cultured lymphocytes (14.6%, including 10% centromere-positive cells), which additionally indicates high mitotic instability of r(8). Thus, r(8) is characterized by mitotic instability, and, obviously, the degree of instability is different in various types of cells and tissues. Moreover, cells with the 45,XY,−8 karyotype tend to restore the 46,XY karyotype by amplifying the remaining normal homologue of chromosome 8, i.e., the paternal homologue is doubled—UPD(8)pat.

The most common features in patients with r(8) are short stature, microcephaly, nonspecific dysmorphic features, intellectual disability, and ADHD (Table 1). Developmental delays were mentioned for four of the eight patients. The index patient demonstrated the following features described in other patients with r(8): low birth weight and length, microcephaly, pectus excavatum, hypotonia, and language delay. His facial dysmorphic features were nonspecific, except for micrognathia (Table 1). In addition, the boy had symptoms that have not been described in patients with r(8) or were opposite to those previously described: some dysmorphic features (e.g., elongated face, which appeared by 2.5 years of age; short and broad philtrum, thick lips, and bushy eyebrows), restricted facial movements, tall stature, and conjunctivitis.

There are seven patients with UPD(8)pat described in the ChromosOmics Database [45]. For two probands clinical symptoms were unavailable. For the remaining five different pathological phenotypes were described: lipoprotein lipase deficiency (patient 08-WpU-N/1-1), Asperger syndrome and attention deficit disorder (patient 08-WpU-N/2-1), congenital adrenal hyperplasia (patient 08-WpU-N/3-1), spinal muscular atrophy with progressive myoclonic epilepsy (patient 08-WpU-N/4-1), and hereditary motor and sensory neuropathy, type Lom (HMSNL), CMT4D (patient 08-WpU-N/5-1). In our patient, there were problems with fine motor skills, diffuse muscle hypotonia and moderate proximal muscle weakness. According to electroneuromyography (ENMG), spinal muscular atrophy (SMA) or atypical SMA could not be excluded and the patient requires further dynamic observation.

In 2008, Barel and colleagues described for the first time a large family with a *KCNK9* imprinting syndrome characterized by congenital central hypotonia, severe feeding difficulties, DD/ID, characteristic dysmorphic features (elongated face, downturned open mouth, and thick lips), and hyperactivity (Table 2). This syndrome developed due to the mutations c.770G>A and c.770G>C in the expressed maternal allele of the *KCNK9* gene located at 8q24.3. Both mutations were predicted to change the protein (p.Gly236Arg) and fully abolish the activity of the potassium channel when acting as both a homodimer and a heterodimer [17]. The authors expected that the physiological impact of a dominant loss-of-function mutation might exceed that of a completely null mutation because the nonfunctional protein might affect other protein partners (e.g., K_2P_3.1 and HCN2). However, some pathogenic effects from completely null mutations certainly occur. Therefore, the situation in which maternal chromosome 8 is lost during embryogenesis for any reason (e.g., when it is in a ring form) in a subset of the nerve cells might lead to the development of a relatively mild or atypical form of BBS.

In the index patient, several clinical features typical of BBS could be suggested, i.e., hypotonia, feeding difficulties, developmental delay, elongated face, and thick lips. For a more detailed comparison, see Table 3. Moreover, ENMG could not exclude one of the genetic variants of SMA or atypical SMA in the given patient. Taking into account a heterogenic nature of SMA, it should be noted that this clinical feature is also characteristic of BBS. Significantly, some other features overlap with those observed in patients with r(8) and motor problems were also described in two patients out of five with UPD(8)pat. It is worth emphasizing that in all cases where the origin of r(8) was determined, it was maternal; this is true in the patient described here as well. In these patients, an additional cell line with monosomy 8 or even a normal karyotype was observed, which allows to assume that in some cells, there was a single or doubled paternal chromosome with inactive *KCNK9* (UPD(8)pat). Thus, it is not surprising to see, at least to some degree, the clinical phenotype of BBS in patients with r(8).

In order to exclude three known mutations in the *KCNK9* gene responsible for BBS (c.770G>A, c.770G>C, or c.710C>A) [17,19] the targeted next-generation sequencing of DNA sample obtained from peripheral blood lymphocytes of our patient was performed. The analysis did not identify either of the mutations reported in literature.

Therefore, we can suggest here a possible mechanism for imprinting syndrome origin. Let us consider this mechanism using the example of our patient with ring chromosome 8. A ring chromosome 8 with an imprinted gene was carried to a zygote by an oocyte. The ring chromosome is characterized by mitotic instability. During the early stages of embryogenesis, cells divide rapidly, which may lead to the loss of the ring chromosome and the appearance of cells with paternal monosomy 8. However, monosomy is an unfavorable karyotype for cell survival and function; therefore, the duplication of the remaining normal homologue occurs to stabilize the karyotype leading to UPD(8)pat. Remarkably, the same sequence of events was reported in 2014 by Bershteyn and colleagues in induced pluripotent stem cell (IPSC) lines derived from the skin fibroblasts of a patient with Miller–Dieker syndrome due to 17p13.3 deletion arisen during r(17) formation [46]. Taking into account some crucial common features in the biology of pluripotency between IPSCs and embryonic stem cells (ESCs), the induction of chromosomal instability in cells with ring chromosomes may be feasible at the ESC stage. If a ring chromosome carries an active imprinted allele, then after ring elimination, or even after karyotype rescue by duplication of the intact homologue, there will be no expression of an imprinted gene in a cell at all. Thus, chromosomal mosaicism may appear and lead to a mild or atypical manifestation of an imprinting syndrome. The development of a mosaic imprinting disorder through the loss of a mitotically unstable ring chromosome has, to our knowledge, been mentioned previously in the literature only once, by Robinson and colleagues, when they discussed a patient with PWS and non-mosaic r(15) of paternal origin in blood culture [20].

In conclusion, we present a patient with a 46,XY,r(8)/45,XY,−8 karyotype in whom some clinical features are consistent with BBS phenotype. One can suggest that a mosaic imprinting syndrome could develop due to the loss of mitotically unstable r(8) in a portion of target cells during early embryogenesis. Cell models based on IPSCs with r(8) chromosome [47] theoretically can be useful to measure allele-specific gene expression in derivative specialized cells, but at a present time these studies are significantly restricted due to imprinted disturbances arising during cell reprogramming. Notably, successful attempts have been described in the literature to treat BBS with mefenamic acid (MFA) [18]. Thus, we would like to pay attention of geneticists and pediatricians who may counsel patients with ring chromosomes containing imprinted genes that loss of the ring chromosome with active allele may be consistent with a mild or atypical case of an imprinting syndrome.

## Figures and Tables

**Table 1:** Symptoms in patients with ring chromosome 8.

	Pfeiffer and Lenard [21]	Hamers and van Kempen [22]	Mingarelli et al. [23]	Verma et al. [24]	Bonet et al. [25]	Le Caignec et al. [2] Patient III-1, 6.5-Year-Old Boy	Le Caignec et al. [2] Patient II-1, Mother of Patient III-1, 30 Years Old	Gradek et al. [26]	Index Patient, This Study
Karyotype	46,XY,r(8)/ 46,XY?	46,XY,r(8)[117]/ 46,XY[1]/ 45,XY,−8[1]/ 47,XY,r(8),+r(8) [1]—lymphocytes 46,XY[64]/47,XY,r(8),+r(8) [5]/?[3]—fibroblasts	46,XX,r(8)	46,XX, r(8), inv(7)	46,XY,r(8)/ 45,XY,−8/ 47,XY,r(8), +r(8)	46,XY,r(8)(p23q24.3) [24]/45,XY,−8[2].ish r(8)(8ptel+,8qtel+)	46,XX,r(8)(p23q24.3) [22]/45,XX,−8[2]/ 47,XX,r(8)(p23q24.3), +r(8)(p23q24.3)[1].ish r(8)(8ptel+, 8qtel+)	9 years, lymphocytes: 46,XY,r(8)[27]/ 46,XY, tan r(8)[1]/ 45,XY,−8[2]; 12 years, lymphocytes: 46,XY,r(8)[27]/ 46,XY, tan r(8)[2]/46,XY[1]; 19 years, lymphocytes: 46,XY,r(8)[23]/ 46,XY[7]; 12 years, skin fibroblasts: 46,XY,r(8)[29]/45,XY,−8[3]/ 46,XY[18]	1 year 4 months, lymphocytes: 46,XY,r(8)(p23q24.3)[27]/ 45,XY,−8[3]
Ring origin	−	−	−	−	−	Maternal	Maternal	Maternal	Maternal
Family history	−	No abortions or stillbirths	−	−	−	−	No previous history of miscarriage	Two normal siblings	First pregnancy ended with miscarriage
Intrauterine growth retardation	−	−	−	−	−	+	−	−	−
Birth weight	−	2770 g (10th centile)	−	2000 g (<3rd centile)	−	1350 g (5th centile)	−	3170 g (25th centile)	2720 g (3–10th centiles)
Body length	−	47 cm (10th centile)	−	−	−	38.5 cm (5th centile)	−	50 cm (50th centile)	48 cm (10th centile)
Head circumference	−	−	−	29 cm (<3rd centile)	−	27.5 cm (<3rd centile)	−	33 cm (5–10th centiles)	31 cm (3rd centile)
Gestational age at birth	−	(13 days after term)	−	40 weeks	−	Premature	−	At term	38 weeks
Developmental delay	−	+	−	+	−	+	−	+	+
Short stature	+	+	+	−	+	+	+	+	−
Microcephaly	+	+	+	+	+	+	+	+	+
Facial dysmorphia	Turricephaly, flat occiput, hypotelorism, dental anomalies, micrognathia	Dolichocephaly, prominent occiput, bilateral strabismus, epicanthic folds, asymmetric ears, thin upper lip, gothic palate, asymmetry of the upper dental arch, micrognathia	Slightly sloping forehead, flat face with prominent glabella, upward slanted palpebral fissures, hypertelorism, bilateral epicanthic folds, flat nasal bridge	Prominent nose, high arched palate, low-set left ear	Hypotelorism, bilateral epicanthic folds, long philtrum, narrow palate, low-set ears, thin lips, micrognathia	Small nose, anteverted nostrils, long philtrum, thin upper lip	−	Brachycephaly, antimongoloid slant, bilateral epicanthus, prominent ears	High forehead, flat sloping occiput, short neck, abnormal hairline, preauricular tag of the left ear, bulbous nose, smooth philtrum, macrostomy, thick lips, high palate, irregular teeth growth, micrognathia
Other anomalies	Unilateral cryptorchidism, coxa valga	Clinobrachydactyly of fifth fingers, pectus excavatum, scapulae alatae, long thorax, amblyopy, camptodactyly of both fifth fingers, hernia inguinalis (bilateral and operated), dislocation of the hip, hypertelorism of the nipples, sacral dimples, dimples dorsal of the elbows, cutis marmorata	Brachydactyly of the fifth fingers	Bilateral equinovarus feet, limitation of dorsiflexion of the feet and joints, hyperreflexia	Clinobrachydactyly of the fifth fingers	Clinobrachydactyly of the fifth fingers, amblyopy	−	Broad neck, hypertelorism of the nipples, mild brachydactyly of the fifth fingers	Broad fingers, proximally placed thumb (bilateral), thickening of the distal phalanx of the hallux (bilateral), dysplastic nails, pectus excavatum, pes planovalgus
Speech and language delay	−	−	−	No speech at 13 years	−	+	−	+	+
Learning difficulties	−	−	−	−	−	+	Mild	+	n/a
Intellectual disability	Mild	Severe	Mild	Severe	Moderate	Mild	Normal intelligence	Borderline to mild (IQ 70)	n/a
Behavior	−	−	Hyperactivity, pleasant personality	Hyperactivity, self-stimulatory behavior, no social contact, unable to feed herself	Pleasant personality, attachment for people and things with unrestricted affect	ADHD	−	ADHD, kind, confident, empathic, socially well adjusted	−
MRI, CT, echoencephalography	−	−	−	−	−	Focal pachygyria with decreased sulcation and a thickened cortex in the frontal and occipital areas bilaterally, mild-amplitude rhythmic activity	−	Normal	Ventriculomegaly
Feeding	−	Difficulty in the neonatal period	−	−	−	−	−	Regurgitation, vomiting, poor appetite	Mild dysphagia
Seizures	−	+	−	−	−	−	−	−	−
Recurrent infections	−	+	−	−	−	−	−	−	−
Hypotonia	−	+	−	−	−	−	−	+	+
Decreased fetal movement during the pregnancy	−	−	−	+	−	−	−	−	−
Constipation	−	−	−	−	−	−	−	+	−

**Table 2:** Symptoms in patients with Birk-Barel syndrome.

	Barel et al. [17] 15 Members of the Family	Graham et al. [18] Patient 1	Graham et al. [18] Patient 2	Graham et al. [18] Patient 3	Graham et al. [18] Patient 4	Sevida et al. [19]
Mutation	Missense mutation 770G>A in exon 2, replacing glycine at position 236 by arginine (G236R), in *KCNK9*	*De novo* c.706G>C mutation (pGly236Arg) in *KCNK9*	*De novo* c.706G >C mutation (pGly236Arg) in *KCNK9*	*De novo* c.706G>C mutation (pGly236Arg) in *KCNK9*	*De novo* heterozygous c.706G>A; p.G236R mutation in the *KCNK9* gene and m.8902G>A; pA126T variant of uncertain significance in the mitochondrial *ATP6* gene	Heterozygous c.710C>A (p.A237D) in *KCNK9*
Gestational age at birth	−	At term	At term	At term	38.5 weeks	At term
Birth weight	−	2558 g (3–5th centile)	2655 g (10–25th centiles)	2954 g (10–25th centiles)	3302 g (50th centile)	3210 g (25th centile)
Birth length	−	43.2 cm (<3rd centile)	48 cm (25th centile)	53 cm (>90th centile)	52 cm (80th centile)	50 cm (50th centile)
Head circumference	−	33.2 cm (10–25th centiles)	35 cm (50th centile)	34.5 cm (75th centile)	34.5 cm (25th centile)	53.5 cm at 13 years * (50th centile)
Oligohydramnios	−	+	−	−	−	−
Intrauterine growth restriction	−	+	−	−	−	−
Hypoglycemia	−	+	−	−	−	−
Developmental delay	−	+	+	+	+	+
Intelligence	Moderate to severe intellectual disability	−	−	−	−	Border-line intellectual deficit
Behavior and sleep	Hyperactive	Lethargic, fussy, and uncommunicative, sleeping 13–14 h a day	Severe obstructive sleep apnea with both central and obstructive patterns	Fatigability	Interacting well with other people	
Feeding	Severe difficulties in infancy (tube feeding), dysphagia of solid foods until near puberty	Severe feeding problems requiring a gastrostomy tube due to poor sucking and gastroesophageal reflux	Moderate oropharyngeal dysphagia with silent aspiration of thin fluids that required nasogastric tube feedings	Poor feeding	Poor feeding	Weak sucking and episodes of hypoxia during feeding in infancy, dysphagia of liquids with a well-evocable pharyngeal reflex at 17 years
Muscle tone	Generalized hypotonia at an early age followed by weakness of proximal muscles and of the supra- and infrascapular and trapezius muscles later on	Central hypotonia with episodes of spontaneous clonus upon awakening	Congenital hypotonia, diminished facial movements, myoclonic jerks, proximal muscle weakness	Generalized hypotonia, weakness	Mild generalized hypotonia	Generalized muscle weakness, mild cerebellar syndrome, peripheral motoneuron syndrome, areflexia, problems with fine and gross motor skills, mild contractures of triceps surae muscles and decreased physical endurance, hypomimia, bilateral lagophthalmos, tongue fasciculations
Facial dysmorphia	Elongated face with a narrow bitemporal diameter, mild atrophy of the temporalis and masseter muscles, reduced facial movements; flared, bushy, and arched upward eyebrows, downturned eyelids, sparse eyelashes in the inner third of the lower eyelids, congested conjunctivae; protruding ears with a very prominent fold of the crux of the helix and a prominent antihelical fold; high and narrow nasal bridge with a broad nasal tip; extremely short, broad, and thick philtrum; prominent maxillary and premaxillary regions, hypotonia of the mandible, micrognathia, open mouth; thick lips, downturned upper lip (“fish mouth”), a lower lip shorter than the upper lip; narrow, high-arched palate with a full or submucous cleft; large and protruding incisors	Dolichocephaly with bitemporal narrowing, upswept anterior and posterior hair pattern, short philtrum, tented upper lip, V-shaped cleft palate, prominent maxilloalveolar frenulum, small mandible, medially flared eyebrows	Bitemporal narrowing, tented upper lip, high arched palate, and retrognathia	Thin upper lip, downturned open mouth, broad alveolar ridges, cleft soft palate, micrognathia/retrognathia	A high and broad nasal bridge, a generous mouth with downturned corners	Slightly elongated face, cleft palate, micrognathia, tented upper lip, short philtrum, low-set ears
Other abnormalities	Narrow, elongated neck, trunk, and feet; mild joint contractures of the hips, elbows, phalanx, and feet; pilonidal dimple or sinus	Tapered fingers with prominent fetal fingertip pads, bridged transverse palmar flexion creases, joint laxity	Sacral dimple, extradural lipoma	Patent foramen ovale, sacral dimple, phimosis, small thumbs, soft doughy hands, small feet with high arches, dorsiflexed toes, tremor-like movements of the arms with intention and mild head bobbing, especially when tired	Dextroscoliosis in the thoracic region of 10–15°	−
Speech	Dysphonic	A word, “mama”, at age 19 months under treatment with mefenamic acid	Mainly vowels	−	Several words	Dysarthria, dysphonia
Electromyography and muscle biopsy	Muscle biopsy: compatible with spinal muscular atrophy	−	Electromyography of the right biceps and quadriceps suggested a generalized myopathy, and muscle biopsy showed mild variation in fiber size with a few perivascular mononuclear inflammatory infiltrates. Immunostaining with monoclonal antibodies to sarcoglycans revealed diffuse, slightly reduced sarcolemmal staining of delta-sarcoglycan, patchy reduction in β-sarcoglycan	Normal muscle ultrasound	−	EMG revealed signs of primarily axonal peripheral pure motor neuropathy. The pattern on EMG was similar to the pattern observed in spinal muscular atrophy. A muscle biopsy performed at the age of 18 months showed signs of neurogenic transformation with mosaic selective atrophy of type 2 fibbers and hypertrophy of type 1 fibers
Hyperinsulinism	−	+	−	−	−	−
Markedly diminished tearing upon crying	−	+	+	+	−	−

* Pay attention that head circumference for the patient by Sevida et al. was measured at 13 years unlike the rest patients for whom it was measured just after birth.

**Table 3:** Symptoms in the index patient and patients with the maternal ring chromosome 8 common to Birk-Barel syndrome.

Clinical Features of Birk-Barel Syndrome	Patients with Maternal r(8), [2,26]	Index Patient
Low weight at birth	+	+
Short length at birth	+	+
Microcephaly at birth	+	+
Developmental delay/intellectual disability	+	+
Elongated face	−	+
Bushy eyebrows	−	+
Long eyelashes	−	+
Conjunctivitis	−	+
Abnormal ears	+	+
Short philtrum	−	+
Micrognathia	−	+
Feeding problems	+	+
Reduced muscle tone	+	+
Restricted facial movements	−	+
Abnormal speech	+	+

## Supplementary Materials

The following are available online at https://www.mdpi.com/2073-4425/11/12/1473/s1, Table S1: Primers used for real-time PCR and fluorescence in situ hybridization (FISH)-probe synthesis. Table S2: Primers and PCR conditions used for targeted massive parallel sequencing of two exons of *KCNK9* gene.
