# Supplementary material for: 46,XY,r(8)/45,XY,−8 Mosaicism as a Possible Mechanism of the Imprinted Birk-Barel Syndrome: A Case Study
Source: Genes (Basel). 2020 Dec 9;11(12):1473. doi: 10.3390/genes11121473 (PMC7763634; doi:10.3390/genes11121473)
Supplement: Supplementary file 1 [file genes-11-01473-s001.pdf]

Table S1. Primers used for real-time PCR and fluorescence in situ hybridization (FISH)-probe synthesis.

Region	Primer	Sequence	Analysis
5q13.3	*HEXB* F	5′-CCGGGCACAATAGTTGAAGT-3′	Real-time PCR
	HEXB R	5′-TCCTCCAATCTTGTCCATAGC-3′	
9p21.2	*LINGO2* F	5′-TGAGGTTGGGTTTGTCATCA-3′	
	LINGO2 R	5′-CTGCCCTCGAAGGTGTCTAC-3′	
8p23.3-p23.1	*XKR5* R	5′-GGTTGGGCGGGTACTCCT-3′	
	XKR5 F	5′-GGTTACGGTTCAGGCAAAAC-3′	
8p23.3-p23.1	*UNC5D*_FISH1	F 3′-GCCATGACCAACCCGTTCAT-5′	FISH
	*UNC5D*_FISH1	R 3′-GGGTGGCAAACATCGCTACA-5′	
	*UNC5D*_FISH2	F 3′-TGGTGGTCTGCAAGTACCCA-5′	
	*UNC5D*_FISH2	R 3′-AGGGCACTTGGAGGGTCAAT-5′	
	*UNC5D*_FISH3	F 3′-CAGGTCTTGCGGTCTACGTT-5′	
	*UNC5D*_FISH3	R 3′-CCCACCATTGAGAGGAGCTG-5′	
	*UNC5D*_FISH4	F 3′-GGGAAAGGGAAAGGGTCGTA-5′	
	*UNC5D*_FISH4	R 3′-ATTCACATCGCCTCTTGTGC-5′	
	*TUSC3*_FISH1	F 3′-CACGCTATATTGGAAAGTGTGGT-5′	
	*TUSC3*_FISH1	R 3′-TTACACAGCTCAGCCACAATTAG-5′	
	*TUSC3*_FISH2	F 3′-TTGACTCAGAAAGCAATGAAGCG-5′	
	*TUSC3*_FISH2	R 3′-CAATCTTCCAGACTTCCAGACCA-5′	
	*TUSC3*_FISH3	F 3′-CTGTAGAGCCAGACTGTCAATGT-5′	
	*TUSC3*_FISH3	R 3′-GTATCCTGTCTGCTTGTTGCTTG-5′	

Table S2. Primers and PCR conditions used for targeted massive parallel sequencing of two exons of the *KCNK9* gene.

Primer	Sequence	PCR Conditions	Size, bp
*KCNK9*_Seq_ex1-F	5′-GCCACTTCGCAAACTTTTCCTC-3′	94 °C 4 min<br>10 cycles:<br>94 °C 20 s<br>58.5 °C 30 s<br>68 °C 13 min<br>20 cycles:<br>94 °C 20 s<br>58.5 °C 30 s<br>68 °C (13 min<br>+10 s each next cycle)<br>hold at 4 °C	1331
*KCNK9*_Seq_ex1-R	5′-CCAGAGAGGTAGTAAGTGTAAGGC-3′		
*KCNK9*_Seq_ex2-F	5′-AAGAAATCAAGCTACCCTGCTAAC-3′	94 °C 4 min<br>10 cycles:<br>94 °C 20 s<br>60 °C 40 s<br>68 °C 13 min<br>20 cycles:<br>94 °C 20 s<br>60 °C 40 s<br>68 °C (13 min<br>+10 s each next cycle)<br>hold at 4 °C	3013
*KCNK9*_Seq_ex2-R	5′-CTGAGTATAGCCAAGGTCCATTTC-3′		
